# Successional Distance between the Source and Recipient Influence Seed Germination and Seedling Survival during Surface Soil Replacement in SW China

**DOI:** 10.1371/journal.pone.0079125

**Published:** 2013-11-01

**Authors:** You-xin Shen, Lei Gao, Xue Xia, Yuhui Li, Huilin Guan

**Affiliations:** 1 Key Laboratory of Tropical Forest Ecology, Xishuangbanna Tropical Botanical Garden, Chinese Academy of Sciences, Menglun, Yunnan, P.R. China; 2 Restoration Ecology Group, Xishuangbanna Tropical Botanical Garden, Chinese Academy of Sciences, Kunming, P.R. China; 3 Yunnan Normal University, Kunming, P.R. China; DOE Pacific Northwest National Laboratory, United States of America

## Abstract

Adding propagules (source) to a degraded site (recipient) is a common way of manipulating secondary succession to restore diversity and services formerly provided by forests. However, heretofore no study has considered the effect of “successional distance” between source and recipient site. Four sites in the Shilin karst area of SW China were treated as different states along a secondary successional sere: grass, shrub, young secondary forest, and primary forest. Ten 1 m ×1m soil quadrats in the grass, shrub and young forest sites were replaced with 10 cm deep soil sources from corresponding later successional stage(s) in January 2009. Woody plant seed germination was monitored in the first year and seedling survival was monitored until the end of the second year. At the end of 2010, 2097 seeds of woody plants belonging to 45 taxa had germinated, and 3.9% of the seedlings and 7.8% of the species survived. Germination of most species was sensitive to ambient light (red, far-red, R:FR ratios, photosynthetically active radiation). Soil source and recipient site had a significant effect on the total number of seeds and number of species that germinated, and on the percentage of seedlings that survived through the end of the second year. Closer successional stages between recipient site and soil source had higher seed germination and seedling-survival percentages. However, a transition threshold exists in the young forest state, where seeds can germinate but not survive the second year. Our results, although based on an unreplicated chronosequence, suggest that successional distance between soil sources and recipient sites affect forest recruitment and restoration in degraded karst of SW China.

## Introduction

Worldwide demands for practices to restore diversity and services formerly provided by forests are increasing. These practices include removing human disturbance (i.e. fire, grazing, and timber or fuel-wood harvesting) to facilitate natural succession, and human intervention to accelerate and influence the successional trajectory of recovery [1–3]. Natural recovery in degraded ecosystems may take decades, and human intervention is required to accelerate recovery processes [4]. Seed and establishment limitations usually restrict plant recruitment at early stages in the plant life cycle, which in turn influence the turnover of species during vegetation succession [5,6]. Therefore, various strategies and technologies have been designed to increase the availability of seed sources and/ or to manipulate site availability of degraded sites in order to direct degraded system development along a desired trajectory [1]. However, interactions between added seeds and recipient site strongly influence the selection of seed sources appropriate for degraded systems, or the selection of manipulations on degraded sites to match added sources. This brings uncertainty to decision making about restoration pathways.

Adding seeds via the soil seed bank (SSB) or from collected seeds [7,8] is a common way to eliminate seed limitation and increase propagule availability in degraded sites. When seeds of a species are added to a recipient site where the species is known to be absent, two outcomes may be observed. If seeds germinate, it means that the site can support seed germination and the absence of this species is at least partially limited by seed availability at this type of site. In contrast, the lack of seed germination suggests a site can not support seed germination, and a regeneration niche for this species is absent. Turnbull et al. [9] reviewed the literature on seed addition and found that 53% of the cases reviewed were limited by seed availability, and in nearly half a positive interaction between recipient site and the added seed sources was found. For cases with positive relationships, the contribution of seed sources at the recipient sites varied greatly. Taking the number of recruits obtained per sown seed as the effect size of seed source, Clark et al. [10] recalculated the results of 43 publications on seed additions and found that the majority of species (68% in undisturbed plots and 81% in disturbed plots) had a small effect size (<0.25), and few species (12% in undisturbed plots and 8% in disturbed plots) had a large effect size ( >0.50). The variation in interaction and effect size may be attributed to many reasons, such as disturbance of recipient site, and soil seed bank types [9,10], all of which are attributes of seed-source and recipient-site characters. 

Succession is a key factor in vegetation dynamics [11]. Both the seed source and recipient site can be related to particular states of a successional trajectory. The traditional view of restoration ecology treated the route from degraded state to desired state as an ordered continuum [12], where adding seed sources from a later stage to an earlier stage may directly accelerate succession. Recently, it has been suggested that degraded ecosystems may not always undergo an ordered and gradual development but may undergo nonlinear and threshold transitions [13,14]. According to this state and transition model, the successional trajectory has a range associated with it, and some transitions and states are undesirable and may cross undesired thresholds. Thus, adding sources of a later stage to an earlier stage of restoration becomes more complicated, and results of seed addition are less predictable. Whether succession follows the traditional linear route or has a range, the state of a degraded site does have a “distance” to the latest successional state, as well as to the state of the seed source. This “successional distance” may determine the reaction and its extent of seed addition. The output of experiments of top soil relocation on mine slag (i.e. on coal mine [15], on lead/zinc tailings [7]) showed that later successional species were always absent when surface soil from a later successional stage was relocated to substrates at a very early successional stage. This suggests a large successional distance between the seed source of later successional stages of a plant community and a highly degraded recipient site in an early successional stage can strongly influence the outcome of the top soil relocation. Thus, this particular soil source may not be the most appropriate remediation for such systems.

 However, no study has considered the effect of successional distance of the source and the recipient site on recruitment and restoration. Surface soil of later successional stage communities contains seeds representing those successional stages, the persistent seeds of plants of earlier successional stages and a superior germination substrate [16]. Replacing soil from an earlier successional stage with that of a later stage provides intact seed sources of the later stage and thus combines aspects of the source and recipient sites. In the present study, surface soils at an earlier successional stage were replaced with soils from one to three later successional stages. By monitoring germination and seedling survival, we sought to answer the following questions:

1. Does the soil source from a later successional stage favor the establishment and abundance of plant communities at earlier successional stage recipient sites?
2. Does the successional distance between the soil source and recipient site affect seed germination and seedling survival? In other words, does the same soil source have different effects on different earlier successional stage recipient sites? Similarly, do the soil sources from different successional stages have different effects on the same recipient site?

## Material and Methods

### Study sites and sampling procedure

This study was conducted at Shilin Stone Forest Geographical Park (24°38'-24°58'N, 103°11'-103°29'E), Yunnan Province, southwest China, a karst geo-park famous for rugged limestone landscapes. This park is approximately 900 km^2^ with underlying Permian carbonate rocks, of which more than 400 km^2^ has developed into karst and related landforms. Altitudes range from 1600 to 2200 m above sea level. Mean annual precipitation is 968 mm (at 1680 m), 80% of which falls between May and October. Mean annual temperature is 16.2 °C, mean maximum temperature 20.7 °C (July), and mean minimum temperature 8.2 °C (January) [17]. The primary forest on this karst land is an evergreen broadleaved forest mixed with a few deciduous species [18]. Over the past several decades, much of the primary forest has been removed as a result of human activities such as firewood harvesting and clearing of land for agriculture and animal grazing, followed by the establishment of shrub land and grass land in the area. All necessary permits were obtained for field survey and soil replacement experiment from the park authority.

Four sites covered with grass, shrubs, young secondary forest and primary forest were selected for this study. All four sites are located on the same karst rock base, and have similar geographical features. Thus, we suggest the vegetation cover at these four sites can be treated as different states along the secondary successional trajectory. A preliminary study (three 20 m × 20 m plots) showed that there are 633 ± 29 (ind./ha ± SD) stems (DBH ≥3 cm), with DBH = 10.7 cm ± 7.4 (average ± SD) from 24 species at the primary forest site, 375 ± 39 (ind./ha± SD) stems (DBH ≥3 cm), with DBH = 7.1 cm ± 4.6 (average± SD) from 12 species at the young forest site, and there were a few individual (DBH <3 cm) trees that had sprouted at the shrub site. Grasses mixed with a few shrubs dominated at the grass site (Table 1). We treated vegetation at the grass site as successional state 1, the shrub site as state 2, the young forest site as state 3, and the primary forest site as state 4. Thus the “successional distance” was simplified by the state difference between the vegetation of the seed source and recipient in the following experiments. 

**Table 1:** The dominant woody species found in the tree layer and the shrub layer at the four study sites.

Species	Tree layer	Shrub layer
*Cyclobalanopsis glaucoides* Schottky	PF,YF	
*Neolitsea homilantha* Allen	PF,YF	
*Olea yunnanensis* Hand-Mazz	PF,YF	SS
*Pistacia weinmannifolia* J. Poisoon ex Fr.	PF,YF	SS
*Pistacia chinensis* Bunge	PF	
*Albizia julibrissin* Durazzini	PF	
*Carpinus mobeigiana* Hand.-Mazz	PF	
*Myrsine semiserrata* Wall.		PF
*Zanthoxylum scandens* Bl.		PF
*Trachelospermum bodinieri* (Levl.) Woods.ex Rehd.		PF
*Smilax* sp.		PF, YF
*Dalbergia mimosoides* Franch.		PF
*Ficus ti-koua* Bur.		SS, GS
*Diospyros mollifolia* Rehd. et. Wilson.		YF, SS
*Sophora davidii* (Fr.) Komarov ex Pavd.		SS, GS
*Rhamnus leptophylus* Schneid		YF, GS
*Myrsine africana* L.		SS, GS
*Campylotropsis polyantha* (Franch.) A.k.Schindl.		SS, GS
*Spiraea martinii* Levl.		SS, GS

PF: Primary forest; YF: Young forest; SS: shrub site; GS: grass site

### Soil collection and preparation

Soil was collected from the surveyed primary forest, young forest and shrub sites, but not from the grass site because we are only concerned with the effect of soil sources from later successional stages. Thirty 1m ×1m soil sample quadrats in the primary forest site, 20 at the young forest site and 10 at the shrub site were established. In January, 2009, all soil and litter to a depth of 10 cm were collected from each quadrat, put into plastic bags, and transferred to a concrete platform, where soil from each site was combined and hand mixed. Then, the total amount of source soil from each site was weighed and divided evenly into 30 parts from the primary forest site, 20 parts from the young forest site and 10 parts from the shrub site. By mixing and re-dividing the soils, we aimed to reduce the variation caused by the difference among the parts of the same soil sources since most seeds are unevenly distributed on the forest floor. We refer to soils containing the soil seed banks as the ‘soil source’; we used soil sources from the primary forest (state 4), young forest (state 3) and shrub (state 2) sites. 

### Soil replacement and seedling monitoring

Plants, soils, and stones to a depth of 10 cm were removed from 30 1 m ×1m plots in the grass site, 20 in the shrub site and 10 in the young forest site (quadrats in the young forest and shrub sites were reused). We refer to these as recipient site. Soil sources from later successional stages were transferred to a plot at sites with an earlier vegetative successional stage, so that the following soil transfers and successional distance were made:

1. Distance across one state: primary forest to young forest, young forest to shrub, and shrub to grass;
2. Distance across two states: primary forest to shrub, young forest to grass; and
3. Distance across three states: primary forest to grass

We transferred 10 replicates of each soil source at each recipient site, making a total of 30 plots at the grass site, 20 at the shrub site, and 10 at the young forest site. The successional distance between soil source and recipient site may be caused by the soil itself and viable seeds within the soil. All plots in the same site were numbered and randomly selected to receive different soil sources. A map showing the distribution of plots was prepared for future identification. A thin layer of rice straw was placed on each surface treatment to prevent moisture loss. The straw was removed on April 6-10, 2009, before the beginning of the rainy season in May, to monitor germination. Only seedlings of woody species were identified and recorded (herb seedlings were discarded). Seedlings were recorded at half- month intervals from June to September 2009, and then at 1-month intervals from October 2009 to the end of 2010. At each observation time (t) we counted live seedlings (NA_t_) and dead seedlings (ND_t_) of each species in each plot. After recording their number, the dead seedlings were removed to prevent recounting. 

 Red light (R), far-red light (FR), and R:FR ratios at the soil surface in each plot were measured using a 660/730 nm quantum sensor, and photosynthetically active radiation (PAR) was measured with a 400–700 nm quantum sensor (Skye Instruments Ltd, Powys, UK) between 12:00–14:00 on cloud-free days in September 2009, December 2009, and February 2010. Volumetric soil moisture content (%) (VSMC) at a depth of 10 cm was measured with a PR2 sensor (Delta-T, England) at the same time as light measurements were taken. 

### Numerical analyses

The total number of germinated seeds per species, and their survival percentage are considered in this study. We denote TS_t_(x) as the total number of seeds germinated of species x up to time t, NS_t_(x) as the total seedlings of species x at time t, NA_t_(x) as live seedlings of species x at time t and ND_t_(x) as dead seedlings of species x at time t. Thus, the total number of seedlings of a species at a plot at time t was:

$$NS_{t}\left(x\right)=NA_{t}\left(x\right)+ND_{t}\left(x\right)$$

Some new seedlings may emerge, and some seedlings may die at time t+1, thus, NA_t+1_(x) may be greater than, less than or equal to NA_t_(x) (dead seedlings were taken out after recording at time t). Thus, the total number of seeds germinated at time t+1 is:

*TS*_*t*+1_(*x*)=*TS*_*t*_(*x*)+(*NS*_*t*+1_(*x*)-*NA*_*t*_(*x*)) if *NA*_*t*+1_(*x*)>*NA*_*t*_(*x*)(new seedlings emerged)
Or
*TS*_*t*+1_(*x*)=*TS*_*t*_(*x*) if *NA*_*t*+1_(*x*)≤*NA*_*t*_(*x*)( no new seedlings emerged)

Thus, for species x in plot i, the total number of seeds germinated was the TS(x) at the end of 2009, and the percentage of TS_t_(x) to the total number of seeds germinated was the germination rate at time t. The total number of seeds germinated in a plot was the sum of seeds of each species that germinated. 

For most species, germination stopped, and death of seedlings began before the end of 2009. The proportion of NA_t_(x) to the total number of seeds germinated was taken as the percentage survival of species x at time t (SR _t_(x)), and the proportion of live seedlings (species) to the total number of seeds (species) germinated in a plot at time t as the survival percentage of seedlings (species) of this plot. 

Repeated measures ANOVA was performed in the statistical package SPSS 13.0 with recording time as the repeated measures (within subject) factor and recipient site as between-subjects factors to test the significance of light, and soil water content. The total number of seeds, total number of species identified at the end of 2009, survival percentage of the total number of seedlings and the total number of species were used to test differences among the three soil sources and three recipient sites by two-way ANOVA. Where main effects were significant (P<0.05), Duncan’s multiple range tests were used to compare differences in means of seed germination or seedling survival between soil sources or between recipient sites. 

The relationships between R, FR, R:FR, PAR and VSMC and total emerging seedlings of the species and between environment variables and the survival percentage at the end of second year of the species were analyzed with Canonical Correspondence Analysis (CCA) (CANOCO 4.0). Prior to analysis, seed and environmental data were log(x+1) transformed. To examine the significance of the canonical axis of the partial CCA, Monte Carlo permutation tests were conducted with 199 permutations under a reduced model. The results were displayed as a CCA bi-plot of species and environmental variables.

## Results

### Composition and temporal pattern of germination

Two thousand and ninety-seven seeds from 45 woody plant taxa had germinated at the three recipient sites by the end of December 2009: 13 (28.3%) tree species, 17 (37.0%) shrub species and 9 (19.6%) different woody lianas. Tree seeds accounted for 59.3% of the total, of which *Cyclobalanopsis glaucoides*, *Neolitsea homilantha*, *Pistacia weinmannifolia*, *Pistacia chinensis*, *Albizia julibrissin*, and *Olea yunnanensis* had the largest number of germinated seeds. Shrub seeds accounted for 26.0% of the total, of which *Sophora davidii*, *Zanthoxylum scandens*, *Rhamnus leptophylus*, *Dodonaea viscose*, and *Myrsine africana* had the largest number of germinated seeds. Liana seeds only accounted for 14.0% of the total (Appendix). 

A total of 38 species emerged from the primary forest soil sources at all recipient sites, 30 species from the young forest soil sources and 14 species from the shrub soil sources. More seeds and species of tree and woody lianas germinated from forest soil sources (both primary forest and young forest) than from shrub soil source (Appendix). The shrub soil source contained more shrub seeds than forest soil sources. *Cyclobalanopsis glaucoides* accounted for the largest portion of seedlings germinated from the young forest soil source, and *Sophora davidii* dominated the soil seed bank from the shrub soil source. 

**Figure 1:**
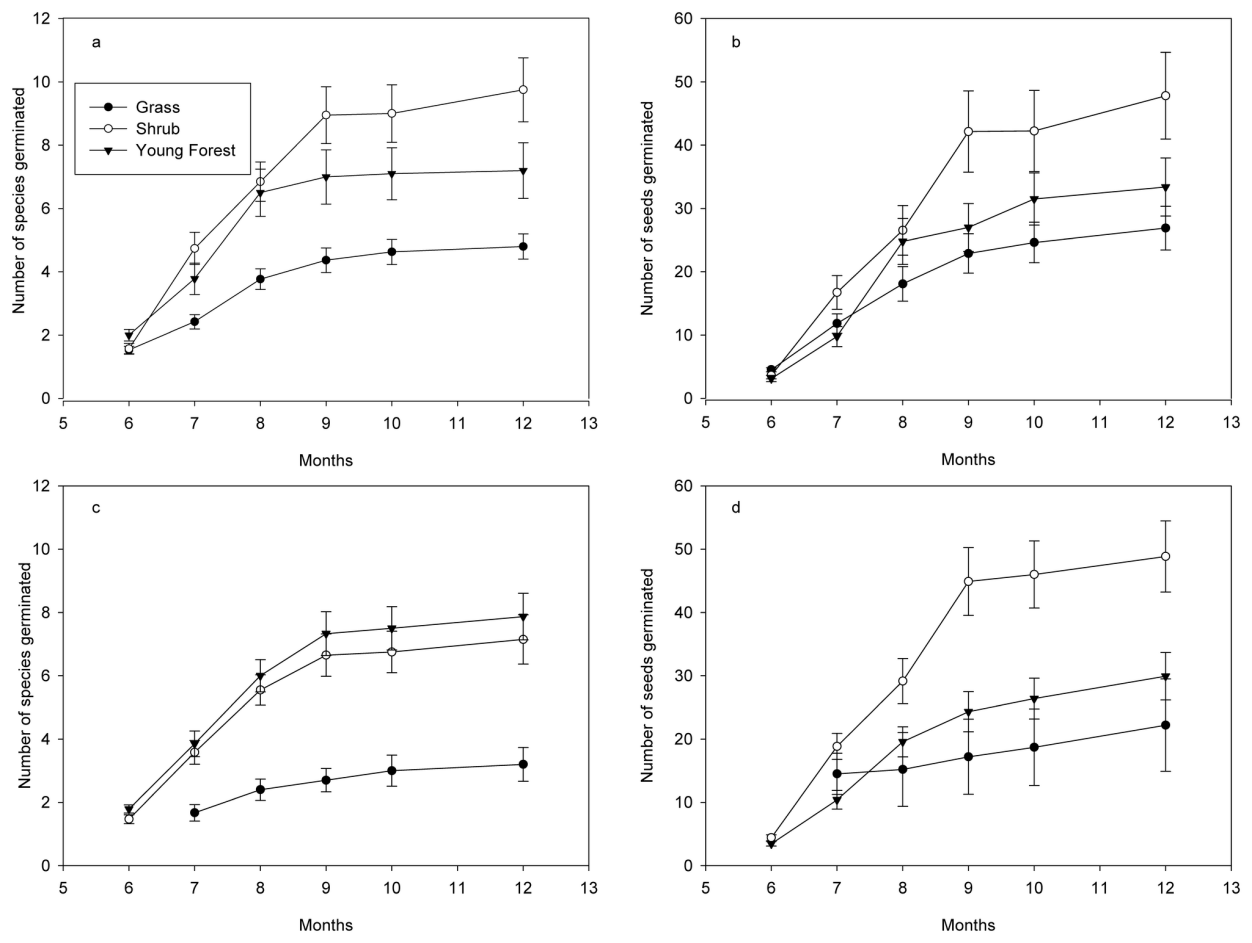
(a) Cumulative number of species (mean ± SE) per m^2^ and (b) number of seeds (mean ± SE) per m^2^ germinated from different recipient sites, and (c) cumulative number of species (mean ± SE) per m^2^ and (d) number of seeds (mean ± SE) per m^2^ germinated from different soil sources at different months after soil (10 cm depth of 1 m ×1m sample) of earlier successional stages were replaced by soil from later successional stages. Grass, shrub, secondary forest and primary forest were treated as different states along a successional sere.

June to September was the main germination period for all three soil source types at each of the three recipient sites. The number of seeds and species increased linearly from June to December 2009 (P<0.001, Figure 1).

### Soil-source and recipient-site effects on seed density and richness

Seed density and richness per square meter varied among soil sources and among recipient sites (Table 2). The recipient site had a significant effect on both the density (F=3.89, P=0.03) and richness (F=9.09, P<0.001) per square meter. Soil source had a significant effect on density (F=4.94, P=0.01), but not on richness (F=2.51, P=0.09). Interactions between the recipient site and soil source were not significant for density (F=0.79, P=0.38) or richness (F=1.59, p=0.21). In the grass plot, fewer woody seeds germinated from primary forest soil than from young forest and shrub soil sources. There were significant differences (P<0.05) between primary forest and young forest soils and between young forest and shrub soils, but not between primary forest and shrub soil sources. Although the difference was not significant between primary forest and young forest soil sources at the shrub site, more seeds germinated from young forest (55.1 m^-2^ ±10.6) than from primary forest soils (40.5 m^-2^ ±8.6). A significantly higher number of species and seeds germinated in the primary forest soil source that was transferred to the shrub site than to the young forest or grass sites. 

**Table 2:** Number of species and of seeds germinated (mean ± SE) per m^2^ until the end of 2009 after soils (10 cm depth of 1 m ×1m sample) of earlier successional stage sites were replaced by soil from later successional stages.

Recipient site	Richness/ Seeds	Soil of State 4	Soil of state 3	Soil of state 2	Average	F(P)
State 1	No. of species	5.5±0.7^aA^	5.7±0.6^a^	3.2±0.5^b^	4.8±0.4	5.31(0.01)
	No. of seeds	15.9±2.4^aA^	42.6±3.5^b^	22.2±7.3^a^	18.9±3.4	8.16(0.002)
State 2	No. of species	10.9±1.5^B^	8.6±1.3		9.8±1.0	1.31(0.27)
	No. of seeds	40.5±8.6^B^	55.1±10.6		47.8±6.9	1.14(0.30)
State 3	No. of species	7.2±0.9^A^				
	No. of seeds	33.4±4.6^B^				
Average	No. of species	7.9±0.7	7.2±0.8			
	No. of seeds	29.9±3.8	48.9±5.6			
F(P)	No. of species	6.49(0.005)	4.0(0.06)			
	No. of seeds	4.76(0.017)	1.25(0.28)			

State 4: Primary forest; State 3: State 2: shrub; State 1: grass

Uppercase letters indicate significant differences between two recipient sites of the same soil source, and lowercase letters indicate significant differences between two soil sources of the same recipient site.

### Recipient- site environment and its effects on germination

**Figure 2:**
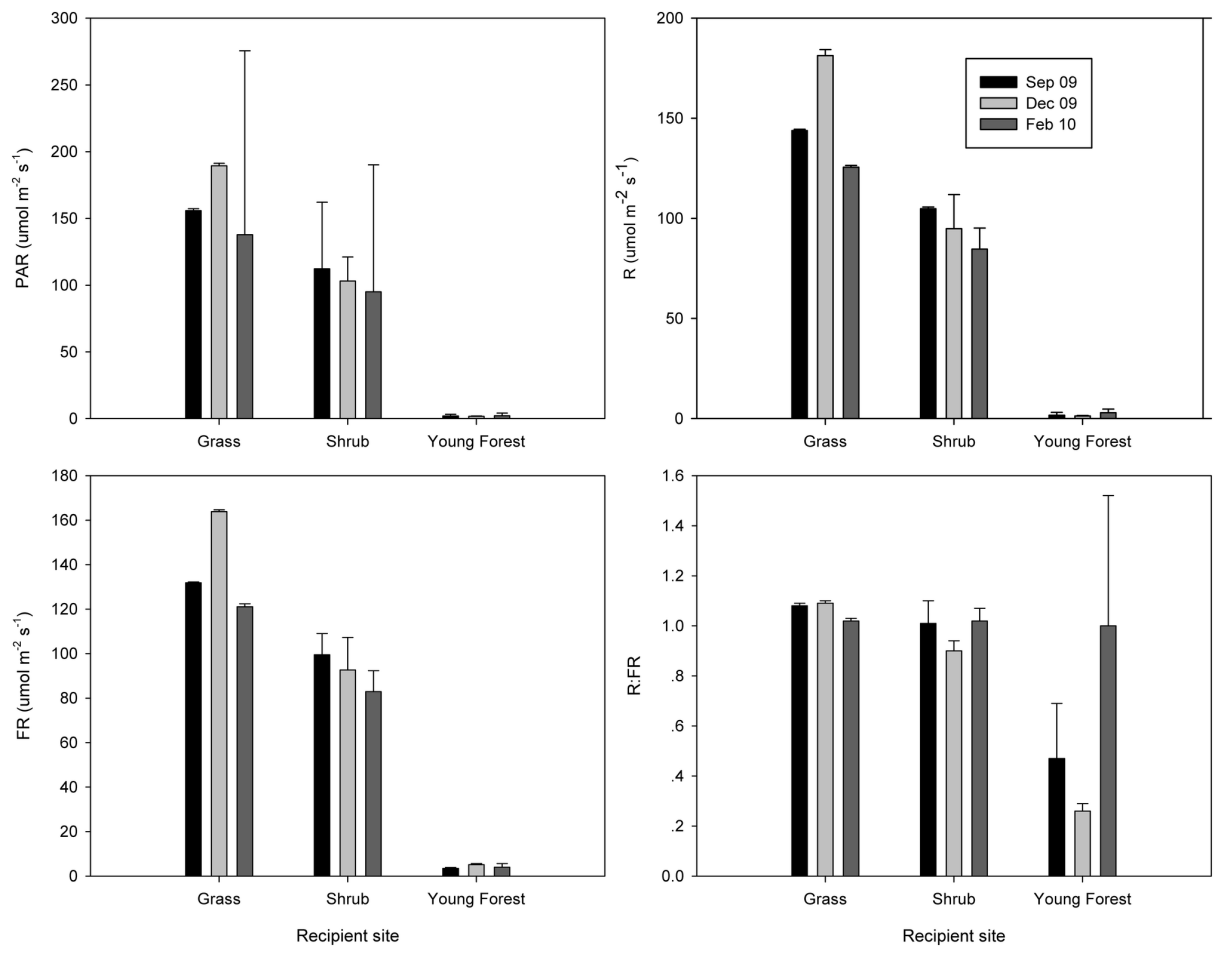
Photosynthetically active radiation (PAR), red radiation (R), far-red radiation (FR), and red/far-red ratio (R:FR) at recipient sites - grass, shrub and young forest - on cloudless days around noon in September 2009, December 2009 and February 2010.

Radiation at the three measurement times (Figure 2) did not differ significantly (repeated measures ANOVA). However, red (F=180.9, P<0.001), far red (F=113.18, P<0.001), red/far red ratio (F=36.56, P<0.001), and photosynthetically active radiation (PAR) (F=103.51, P<0.001) differed significantly among the three recipient sites. Soil water content differed significantly between the two measurement times (Repeated measures ANOVA, F=1067.8, P < 0.001). Where primary forest soil was transferred to the three recipient sites, water content was the lowest in grass and highest in young forest sites, with significant differences found in September 2009. Where young forest soil was transferred, water content was higher at the shrub than at the grass site, however, differences were not significant (Table 3).

**Table 3:** Volumetric water content (mean % ± SE) in September 2009, and February 2010, after soils (10 cm depth of 1m × 1 m sample) in earlier successional stage sites were replaced with soil from later successional stages.

Recipient site	Time	Soil of state 4	Soil of state 3	Soil of state 3	Average	F(P)
State 1	Sep 2009	2.50±0.14^aA^	2.96±0.14^b^	3.21±0.12^b^	2.89±0.82	7.28(0.01)
	Feb 2010	2.84±0.16^a^	3.50±0.17^b^	3.74±0.19^b^	3.36±0.11	7.57(0.001)
State 2	Sep 2009	3.01±0.18^AB^	3.45±0.16		3.23±0.12	3.40(0.07)
	Feb 2010	3.23±0.13	3.53±0.12		3.38±0.90	2.76(0.10)
State 3	Sep 2009	3.10±0.23^B^				
	Feb 2010	3.16±0.11				
Average	Sep 2009	2.87±0.11	3.20±0.11			
	Feb 2010	3.08±0.08	3.52±0.10			
F(P)	Sep 2009	3.01(0.05)	5.41(0.02)			
	Feb 2010	2.49(0.09)	0.01(0.91)			

State 4: Primary forest; State 3: State 2: shrub; State 1: grass

Uppercase letters indicate significant differences between two recipient sites of the same soil source or the same measurement time, and lowercase letters indicate significant differences between two soil sources of the same recipient site at the same measurement time.

**Figure 3:**
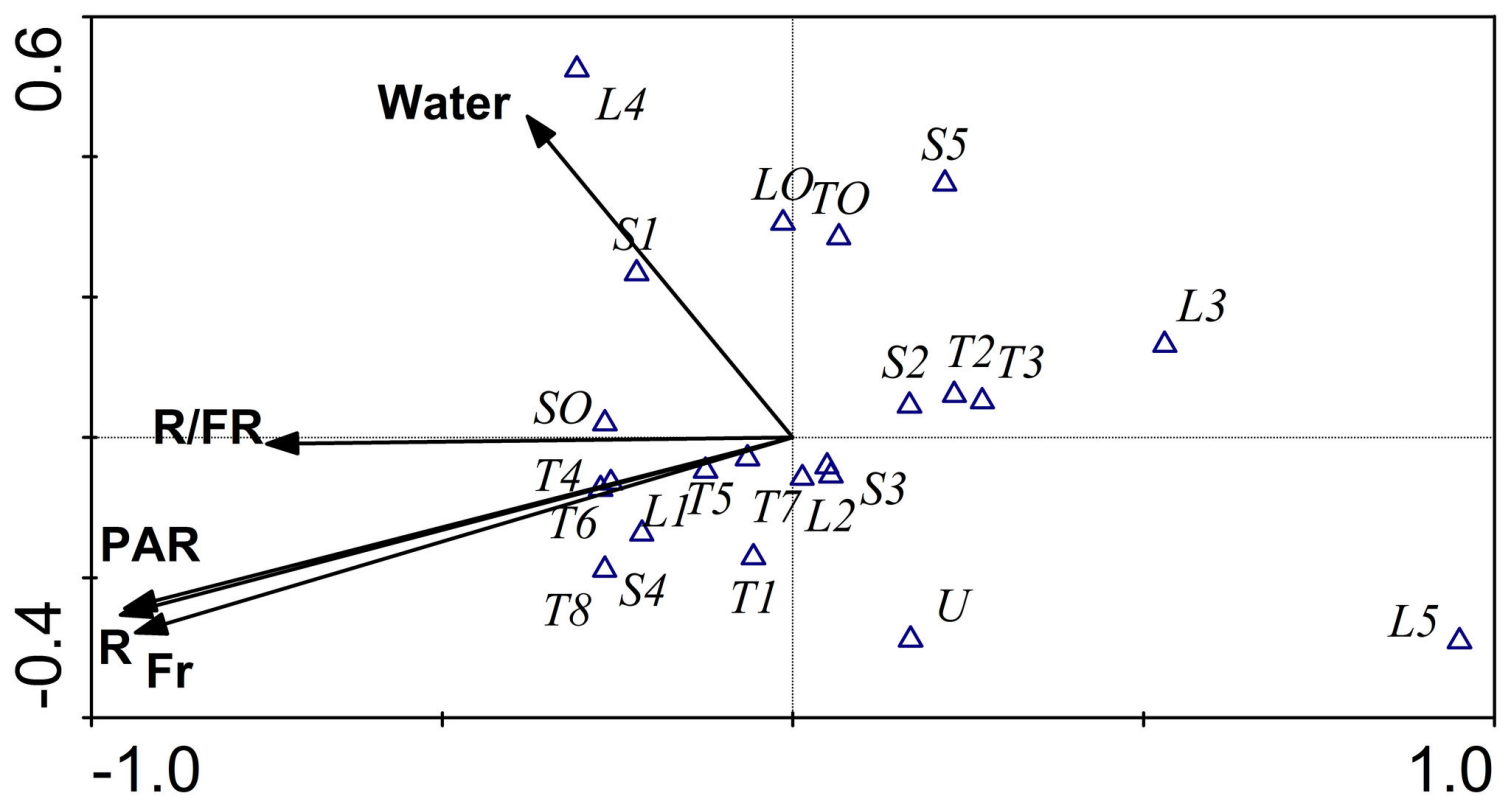
Canonical Correspondence Analysis (CCA) biplot ordination of the number of woody seeds germinated (∆) and environmental variables (arrows). T1-T8 are tree species: *Cyclobalanopsis glaucoides*, *Pistacia weinmannifolia*, *Neolitsea homilantha*, *Pistacia chinensis*, *Albizia julibrissin*, *Olea yunnanensis*, *Neocinnamomum delavayi*, and *Carpinus mobeigiana*; S1-S5 are shrub species: *Sophora davidii, Rhamnus leptophylu*, *Myrsine Africana*,, *Campylotropsis polyantha*, and *Rhamnella martini*; L1-L5 are woody lianas: *Zanthoxylum scandens*, *Smilax* *sp*., *Trachelospermum bodinieri*, *Ficus* *ti-koua*, and *Dalbergia mimosoides*. TO, SO, LO are other trees, shrubs and lianas respectively. U: unidentified species. R: Red light, FR: far-red light; R:FR: R:FR ratio; PAR: photosynthetically active radiation; Water: volumetric soil moisture content .

Five canonical correlations were obtained between seed germination environmental variables R, FR, R:FR ratios, PAR and water content and the total number of seeds that germinated with CCA. The Monte Carlo permutation test (with 199 permutations) both on the first axis (F=4.38, P=0.005) and on all axes (F=1.88, P=0.005) were highly significant. Canonical axis 1 and axis 2 explained 50.7% and 24.5% of the cumulative percentage variance of species-environment relations, respectively. Axis 1 was strongly correlated with PRA (-0.958), FR (-0.952) and R (-0.937). The weighted correlation coefficient between axis 1 and R:FR was also high (-0.750) (Figure 3). These data indicated that light availability increased from left to right on the axis 1. The second axis was strongly correlated with soil water (0.457), indicating that soil water increased along the second axis.

The optima of many species, including six of the eight tree species (T6, T4, T5, T7, T8, T1), two of the five lianas (L1, L2) and two of the five shrubs (S3, S4) were sensitive to the change in light variables PAR, R, FR and R:FR (Figure 3). A higher portion of seed species tended to germinate at the lower to moderate light conditions. The number of seeds that germinated did not increase at higher light levels. On the other hand, only a few species were sensitive to variation in soil water (S1, L4). Two tree species (T2, *Neolitsea homilantha*, T3, *Pistacia chinensis*), two liana species (L3, *Smilax* sp. *L5, Dalbergia mimosoides*), and two shrub species (S2, *Rhamnus leptophylus, S5, Campylotropsis polyantha*) species were not sensitive to light and soil water.

### Soil- source and recipient- site effects on germinant survival

**Figure 4:**
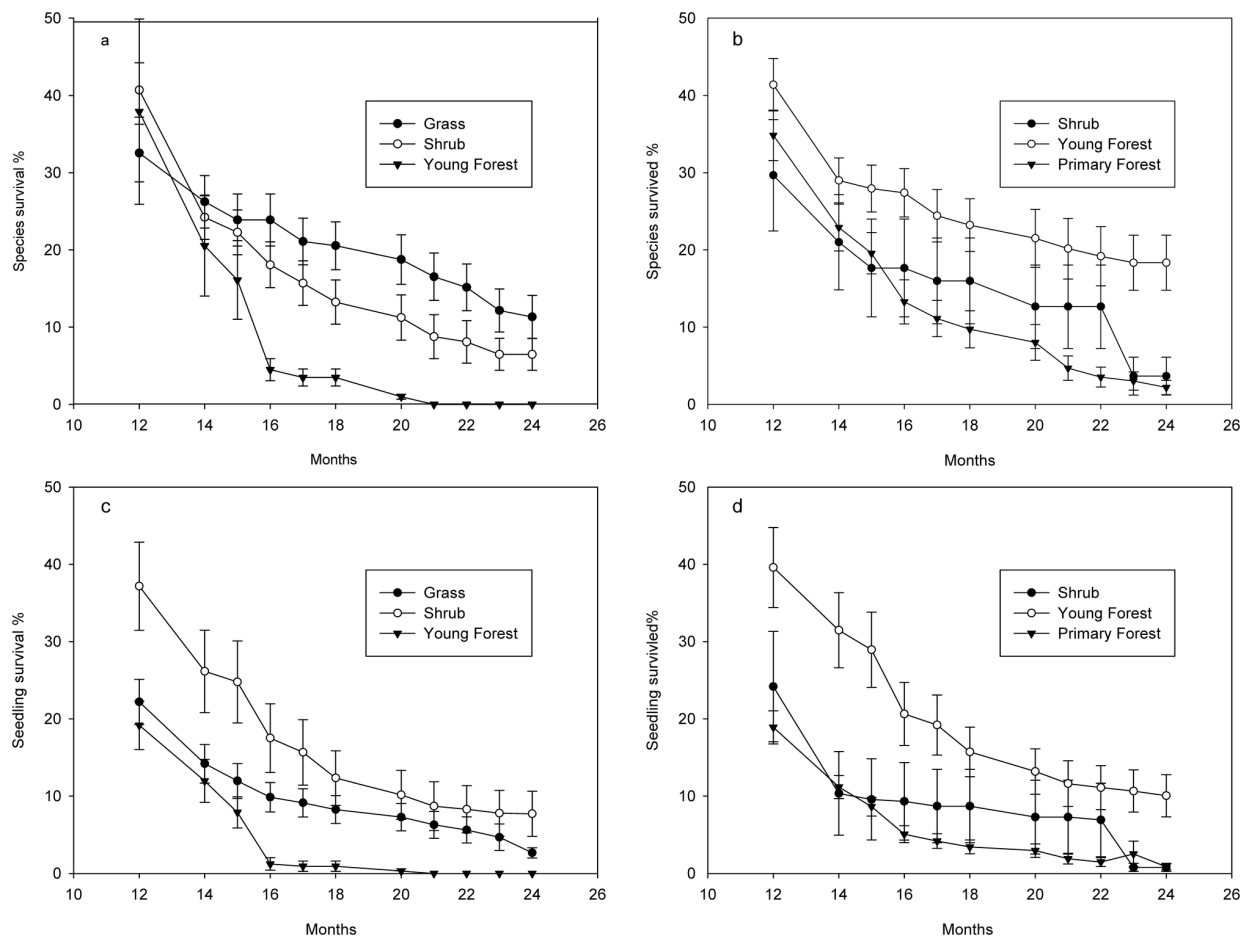
Cumulative survival percentages (mean ± SE) per m^2^ of the number of species (a) at different recipient sites and (b) from different soil sources, and of the number of seedlings (c) at different recipient sites and (d) from different soil sources at different months of the second year after soil (10 cm depth of 1 m ×1m sample) at earlier successional stages were replaced by soil of the later successional stage. Grass, shrub, secondary forest and primary forest were treated as different states along a successional sere.

Survival percentage of seedlings and of species decreased 50% by the end of 2009 (12 months after soil transfers), and this decrease continued through 2010 (Figure 4). Seedlings and species from the young forest soil source had higher survival that those from primary forest soil source for most recording times. Survival percentage was higher at the shrub than at the young forest recipient site. During the dry season of 2010 (12-16 months after soil transfers), the survival percentage decreased quickly (Figure 4). 

At the end of 2010, 3.9% of seedlings, and 7.8% of species were still alive. No seedlings survived at the young forest recipient site. Only 2.65% of seedlings and 11.33% of species survived at the grass recipient sites and 7.72% and 6.49% at the shrub recipient sites (Table 4). Soil source had significant effects on both survival percentage of seedlings (F=8.76, P=0.001) and of species (F=18.09, P<0.001). Recipient sites had significant effects on the survival percentage of species (F=4.75, P=0.02), but not on survival percentage of seedlings (F=1.92, P=0.26). Interactions between the recipient site and soil source were significant for percentage survival of seedlings (F=5.32, P=0.02) and of the number of surviving species (F=6.34, p=0.02). Survival percentages at the grass and shrub recipient sites were significantly higher in the plots with young forest soil sources than in those with primary forest and shrub soil sources. Differences in survival percentages among the three recipient sites with primary forest soil sources were not significant, and neither were the survival percentages in shrub and grass plots with young forest soil sources. On average, survival percentages from the primary forest soil source and percentages from shrub soil source were lower than those from young forest soil sources (Table 4). However, canonical correlation between the final survival percentage at the end of the second year and R, FR, R:FR, PAR and soil water in Feb. 2010 (dry season) was not significant for all axes (F=1.38, P=0.21).

**Table 4:** Seedling survival percentage (% mean± SE) per m^2^ of the number of species and number of seeds at the end of the second year after soil (10 cm depth of 1m × 1 m sample) of the earlier successional stage sites were replaced by soil from later successional stages.

	Recipient site	Soil of state 4	Soil of state 3	Soil of state 2	Average	F(P)
Survival percentage of species	State 1	3.93±2.00a	26.4±5.23b	3.70±2.46a	11.33±2.79	13.65(<0.001)
	State 2	2.68±1.79	10.30±3.40		6.49±2.06	3.95(0.012)
	State 3	0.00				
	Average	2.20±0.92	18.35±3.55			
	F(P)	1.65(0.212)	3.12(0.094)			
Survival percentage of seeds	State 1	1.70±0.97a	5.48±1.28b	0.77±0.52a	2.65±0.66	6.54(0.005)
	State 2	0.82±0.61	14.62±5.01		7.72±2.9	7.49(0.014)
	State 3	0.00				
	Average	0.84±0.39	10.05±2.73			
	F(P)	1.67(0.207)	6.66(0.019)			

State 4: Primary forest; State 3: State 2: shrub; State 1: grass

Lowercase letters indicate significant differences between two soil sources.

*Cyclobalanopsis glaucoides* (16%) and *Olea yunnanensis* (13.2%) had the highest survival percentage. Survival percentage of the other major tree germinants, *Pistacia chinensis*, *Albizia julibrissin*, *Pistacia weinmannifolia* was < 2%. Only a few shrub seedlings survived, including *Rhamnus leptophylus*, *Campylotropsis polyantha*, and liana seedlings from one species, *Dalbergia mimosoides*.

## Discussion

Degraded ecosystems may take an ordered continuum route (in the traditional view) [12] or a nonlinear state and transition route (in the more recent view) [13,14] toward a desired state. Successful recruitment of seedlings at the earlier successional stage site is the initial step to cross states and transitions [19,20]. A large number of woody seedlings that strongly reflected the standing vegetation (Table 1, Appendix) germinated from soil sources collected from both the primary forest and young forest, and even from the shrub site when they were transferred to the earlier successional stage recipient sites. Interaction of soil source and recipient site determined the number of seedlings and of species that germinated and survived in the first and second years. These surviving seedlings and species would contribute to the vegetation succession at the recipient site. 

“Successional distance” between the recipient site and soil source had a significant effect on the seed germination and survival when the soil source was added. Soil transferred from state 3 (young forest) to recipient sites at earlier successional states - state 1 (grass), and state 2 (shrub) - were better than soil sources from state 4 (primary forest) in promoting seedling recruitment because they contained more seeds (Table 2) and provided a greater number of surviving seedlings (Table 4). This contrasts with the conventional procedures of soil seed bank transfer, in which a soil source of the latest successional level is considered to be the best choice. On the other hand, soil sources from state 3 and state 4, had higher numbers of seedlings and species that germinated (Table 2) and those that germinated had a higher seedling survival percentage (Table 4) at the state 2 (shrub) recipient site than at the state 1 (grass) recipient site. Thus, the effect of soil replacement with soil sources from either state 4 (primary forest) or state 3 (young forest) was better at the recipient site of state 2 than at the state 1 site. Most likely, these results showed that the closer the recipient site and the soil source, the higher the seed germination and higher seedling survival. This might explain the absence of later successional stage species when top soil of the higher successional stages is relocated to mine slag at initial successional stages [7,15]. 

Environmental cues that can affect seed germination in natural environments include fluctuations in light quality [21,22], R:FR ratio [23–25] and soil physical condition [26]. Our CCA results also showed that seeds of most species tend to germinate at moderate light and water conditions (Figure 3). At the grass recipient site, light level was strongest (Figure 2), and lowest germination percentages were in primary and young forest soil sources. At the young forest site, light was less intense and seed germination was also low for all plots. At the shrub recipient site, light was moderate, and more seeds geminated in primary forest and young forest soil sources. 

Conditions for survival were more stringent than conditions for germination. In a thorough review on seed addition, Clark et al. [10] found that most species had a survival percentage <25% in the first year. We had a much higher survival percentage (nearly 50%) in all three soil sources at the three recipient sites at the end of the first year (Figure 4). However, these percentages decreased substantially by the end of the second year, and many species had already disappeared from the seedling banks. Therefore, we can say that seed addition of a few selected species in short-term recruitment and succession studies may be misleading. The microsite plays a pivotal role in seedling recruitment [27–29]. Karst soil is highly associated with edaphic aridity and low fertility [30–32]. Even though the CCA result was not significantly correlated with the environmental variables of February 2010 on species survival percentage at the end of the second year, other clues still led us to suppose that environmental factors play important roles on the survival of seedlings. The survival percentages of all combinations during the dry season of 2010 decreased quickly (Figure 4). This may relate to the low soil water content at all three recipient sites in the dry season ( Feb. 2010, Table 3), which was much lower than that at the annual lowest VSMC (13.9%) at 10 cm soil depth in agricultural karst land in Guangxi Province, China [33]. In the first and second years, the 10 cm deep soil of later successional stages strongly affected seedling survival, and thus soil source had significant effects on the survival percentage of seedlings and species. Other environmental factors at the recipient site also contributed to the seedling survival, and thus the interaction effect of soil source and recipient site had significant effects on both survival of seedlings and of species in the second year. Amount of PAR was probably one of the factors determining the survival percentage of seedlings under different canopies [34,35]. All plots at the young forest site were under tree canopies, where PAR was very low, whereas at the grass site it was too intense (Figure 2) for seedlings. Seedling death in the 2009 and 2010 rainy seasons may partially be attributed to damage by mammals, arthropods, and pathogens [36]. 

 A transition threshold controlled by biotic interaction usually exists in the successional process of highly degraded ecosystem [37]. In our study, when soils from state 4 were transferred to the state 3 site, many seedlings of various species germinated in the first year but died by the end of the second year. This result implies that once succession reaches state 3, soil replacement from a later state may not play a role in the successional development of that forest, thus, this state becomes a transition threshold for the succession. Other manipulation tools, such as gap creation, may be needed for survival of seedlings, even for species with high shade tolerance. 

There are weaknesses and potential confounding factors in this study’s design. Soil replacement by a later successional soil source will yield a new combination of seeds and environmental factors, which will in-turn, affect germination and seedling survival. We expected the outcomes of soil replacement to be influenced only by the successional distance between soil source and recipient site. However, other minor factors, i.e. alteration of the soil profile through mixing, and the change of the vertical distribution pattern of seeds may also contribute to seed germination and seedling survival. In addition, a 10 cm deep layer soil-source may not fully function as the original soil in the later successional state. Our conclusions are somewhat weakened because we use an unreplicated chronosequence.

Our findings suggest that soil seed banks in forests in karst areas in SW China, together with their soil substrates can provide woody seedlings for initiating species turnover of degraded forest land. The successional distance between the stage of soil source and the stage of recipient site strongly influence seed germination the first year and seedling survival the second year. These results also suggest that soil sources from secondary forest, and not necessarily primary forest, can satisfy the need for successional manipulation, thus making soil replacement economically and practically preferable since secondary forests are more common and easier to access, and may be spatially closer to the degraded land than primary forest. Consequently, we may save more primary forests. Our results also imply that restoration designs should consider transition threshold states and environmental factors, such as light and soil water. Restoration success at secondary forests could be improved by the implementation of open canopy windows to increase light illumination, and success at grass lands by adding water since both light and soil moisture will affect germination and seedling survival. 

## Supporting Information

Appendix S1 (DOC)
