# Supplementary material for: Successional Distance between the Source and Recipient Influence Seed Germination and Seedling Survival during Surface Soil Replacement in SW China
Source: PLoS One. 2013 Nov 1;8(11):e79125. doi: 10.1371/journal.pone.0079125 (PMC3815137; doi:10.1371/journal.pone.0079125)
Supplement: Appendix S1 — (DOC) [file pone.0079125.s001.doc]

| Species | Family | Soil source of state 4 | | | Soil source of state 3 | | Soil source of state 2 | Total |
| --- | --- | --- | --- | --- | --- | --- | --- | --- |
|  |  | State 3 recipient site | State 2 recipient site | State 1 recipient site | State 2 recipient site | State 1 recipient site | State 1 recipient site |  |
| *Cyclobalanopsis glaucoides* | Fagaceae | 40 | 23 | 2 | 391(112) | 334(15) | 5 | 795(127) |
| *Pistacia weinmannifolia* | Anacardiaceae | 58 | 35(1) | 6 | 5 | 3 |  | 107(1) |
| *Neolitsea homilantha* | Lauraceae | 52 | 30(2) |  | 10 | 7 (1) |  | 99(3) |
| *Pistacia chinensis* | Anacardiaceae |  | 28(1) | 5(1) | 16(1) | 6 |  | 55(3) |
| *Albizia julibrissin* | Leguminosae | 5 | 12 | 31(3) |  | 1 | 3 | 52(3) |
| *Olea yunnanensis* | Oleaceae | 1 | 4 |  | 17(2) | 16(3) |  | 38(5) |
| *Carpinus mobeigiana* | Betulaceae | 7 | 25 |  |  |  |  | 32 |
| *Neocinnamomum delavayi* | Lauraceae |  | 7 |  | 10 | 4 | 4 | 25 |
| Other trees (5 species) |  | 10 | 15(1) |  | 15 | 1 |  | 41(1) |
| *Sophora davidii* | Leguminosae |  | 4 | 6 | 16 | 8 | 162 | 196 |
| *Rhamnus leptophylla* | Rhamnaceae | 41 | 44 | 18 | 5 | 1 | 2 (1) | 111(1) |
| *Myrsine africana* | Myrsinaceae | 11 | 13 | 7 | 16 | 2 | 17 | 66 |
| *Campylotropsis polyantha* | Leguminosae |  | 26 | 4 | 2 | 6(1) | 6 | 44(1) |
| *Rhamnella martini* | Rhamnaceae | 23 | 8 | 9 |  |  | 1 | 41 |
| Other shrubs (12 species) |  | 2 | 25 | 1 | 33(1) | 11 | 15 (1) | 87(2) |
| *Zanthoxylum scandens* | Rutaceae | 10 | 41 | 38 | 3 | 4 | 2 | 98 |
| *Smilax* sp. | Liliaceae | 16 | 49 | 13 |  | 1 |  | 79 |
| *Trachelospermum bodinieri* | Asclepiadaceae | 26 | 5 | 2 | 3 |  |  | 36 |
| *Ficus ti-koua* | Moraceae |  |  | 5 |  | 18 | 5 | 28 |
| *Dalbergia mimosoides* | Leguminosae | 23 |  |  | 4 |  |  | 27 |
| Other woody lianas (4 species) |  | 5 | 5 | 7 | 5 | 3 (1) |  | 25(1) |
| Non-identified species (6 species) |  | 4 | 6 | 5 |  |  |  | 15 |
| Total (45 species) |  | 334 (0) | 405(5) | 159(4) | 551(116) | 426(21) | 222(2) | 2097 (148) |

State 4: Primary forest; State 3: State 2: shrub; State 1: grass
